# Implementation and Performance of a Deeply-Coupled GNSS Receiver with Low-Cost MEMS Inertial Sensors for Vehicle Urban Navigation

**DOI:** 10.3390/s20123397

**Published:** 2020-06-16

**Authors:** Xin Feng, Tisheng Zhang, Tao Lin, Hailiang Tang, Xiaoji Niu

**Affiliations:** 1 GNSS Research Center, Wuhan University, No 129 Luoyu Road, Wuhan 430079, China; focusfeng@whu.edu.cn (X.F.); thl@whu.edu.cn (H.T.); xjniu@whu.edu.cn (X.N.); 2 Beijing Unistrong Science & Technology Co., Ltd., Beijing 100000, China; tao.lin@unistrong.com

**Keywords:** deep couple, GNSS receiver, MEMS IMU, urban environment, vehicle navigation

## Abstract

In urban environments, Global Navigation Satellite Systems (GNSS) signals are frequently attenuated, blocked or reflected, which degrades the positioning accuracy of GNSS receivers significantly. To improve the performance of GNSS receiver for vehicle urban navigation, a GNSS/INS deeply-coupled software defined receiver (GIDCSR) with a low cost micro-electro-mechanical system (MEMS) inertial measurement unit (IMU) ICM-20602 is presented, in which both GPS and BDS constellations are supported. Two key technologies, that is, adaptive open-close tracking loops and INS aided pseudo-range weight control algorithm, are applied in the GIDCSR to enhance the signal tracking continuity and positioning accuracy in urban areas. To assess the performance of the proposed deep couple solution, vehicle field tests were carried out in GNSS-challenged urban environments. With the adaptive open-close tracking loops, the deep couple output carrier phase in the open sky, and improved pseudo-range accuracy before and after GNSS signal blocked. Applying the INS aided pseudo-range weight control, the pseudo-range gross errors of the deep couple decreased caused by multipath. A popular GNSS/INS tightly-coupled vehicle navigation kit from u-blox company, M8U, was tested side by side as benchmark. The test results indicate that in the GNSS-challenged urban areas, the pseudo-range quality of GIDCSR is at least 25% better than that of M8U, and GIDCSR’s horizontal positioning results are at least 69% more accurate than M8U’s.

## 1. Introduction

Autonomous vehicles in urban environments require accurate and timely access to information such as car position, speed and so on [1,2]. In the future, the vehicle-to-vehicle, vehicle-to-infrastructure integrated vehicle networking systems require vehicles to share and interact with navigation information at low latency to complete the overall scheduling and improve traffic efficiency and safety [3,4,5].

Unlike lidar or camera, Global Navigation Satellite Systems (GNSS) is capable of providing high-precision positioning services which are available all-weather, virtually unaffected by light and weather [6,7,8]. Not only can GNSS provides 3D position and speed information for vehicles under an unified reference coordinate system, but also it can provide accurate time information in the areas of navigation and communication [9]. As a result, GNSS is indispensable in an autonomous driving navigation solution. However, in urban areas, GNSS signals are often attenuated, blocked or reflected, which is challenging in GNSS receiver designing [10,11,12,13,14]. If GNSS availability could be improved in challenged environments, the integration navigation solution would reduce the requirements for other sensors’ performance indicators [15,16,17]. As an important sensor of the vehicle navigation solution, the Inertial Navigation System (INS) information can be assisted into GNSS receiver baseband processing to implement a deep couple design, which could eliminate the impact of dynamics caused by vehicle and permit the receiver baseband to work in a quasi-static scene [18,19,20,21,22,23]. In this way the bandwidth can be compressed and the integration time can be lengthened, which will reduce the noise and improve the sensitivity and accuracy of the receiver under dynamics [24,25,26].

The research on GNSS/INS deeply-coupled systems are mainly focused on high dynamics [27,28], anti-jamming [29,30,31,32] for military applications, and sensitivity of civilian applications [33,34,35].

In recent years, GNSS/INS deep couple has been gradually used to improve the measurement performance of GNSS surveying receivers in GNSS-challenged environments [36,37]. In 2008, based on scalar GNSS/INS deeply-coupled architecture, NovAtel and KVH together developed a high precision navigation product [38]. Compared with pure GNSS, its GNSS carrier phase reacquisition speed increases by more than 70%. In 2017, iMAR’s high precision integration navigation product iTraceRT-F402 also applied scalar GNSS/INS deeply-coupled technology to improve its performance of carrier phase reacquisition and tracking [39]. However, high performance INSs are employed in above products, with expensive price and large size, which can not be accepted for autonomous vehicles. In 2014, the authors of Reference [40] implemented the MEMS IMU Mti-G Aided PLL tracking. In 2016, Li et al. also implemented INS PLL based on MEMS IMU [41]. In 2017, the authors of Reference [42] implemented a vector tracking GNSS/INS deeply coupled receiver to improve the performance in GNSS-challenged environments. They developed a maximum-likelihood bit decoding algorithm for bit wipe-off to extend the integration time coherently and achieved good results. The proposed method is sophisticated and not universal enough for receiver. Meanwhile, the vector tracking based design can not track GNSS carrier phase.

According to the description of different deep couple structures in Reference [43], coherent GNSS/INS deep couple structure requires stable tracking of carrier phase to ensure the validity of code tracking, so it is not applicable in GNSS-challenged urban environment. Only non-coherent deep couple is suitable for signal attenuation scenarios. In GNSS-challenged urban environments, however, in addition to attenuation, GNSS signals also suffer from the effect of multipath, frequent interruptions and so on. Although current GNSS/INS deeply-coupled system is capable of improving the continuity of tracking and positioning, it can not improve the accuracy of GNSS observations and positioning in the multipath environments. To improve the quality of pseudo-range and the validity of carrier phase in GNSS-challenged urban environments, we propose a GNSS/INS Deeply-coupled Software-defined Receiver (GIDCSR). A low cost micro-electro-mechanical system (MEMS) inertial measurement unit (IMU) ICM-20602 [44] is employed in the GIDCSR. To enhance signal tracking continuity and positioning accuracy in urban areas, two key technologies are developed in GIDCSR: adaptive open-close tracking loops, INS aided pseudo-range weight control algorithm. To evaluate the performance of GIDCSR, field tests were carried out in the deep urban area near Wuhan University in Wuhan, China. The performance of deep couple before and after applying the two proposed techniques were compared. In addition, since there is no deep coupled product based on MEMS IMU, a commercial racing product-u-blox M8U, a typical tightly-coupled product with a MEMS IMU, was applied. The performances of pseudo-range, GNSS position and integrated position of GIDCSR and M8U are compared.

## 2. GNSS/INS Deeply-Coupled Software-Defined Receiver Implementation

### 2.1. Overview

**Figure 1:**
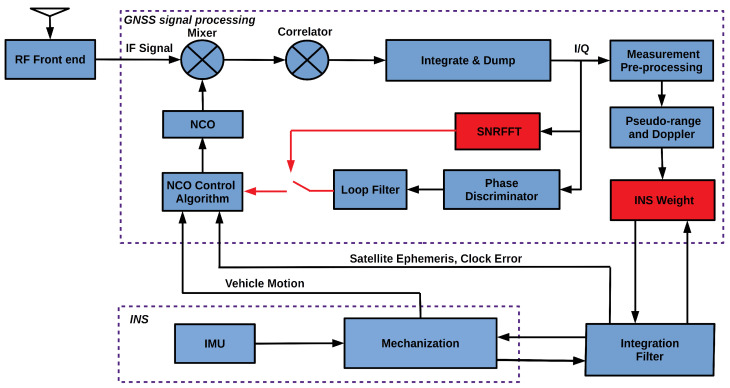
GNSS/INS deeply-coupled software defined receiver architecture.

To improve GNSS measurement and positioning accuracy in GNSS challenged environments, a deeply-coupled receiver with adaptive open-close tracking strategy and INS aided pseudo-range weight control algorithm is proposed based on the non-coherent deep couple structure in [43]. The GIDCSR architecture is shown in Figure 1. The architecture is composed of three blocks which are the GNSS signal processing module, the INS module and the integration filter. GNSS signal processing module is in charge of the processing of GNSS intermediate frequency (IF) signals, producing GNSS observations. INS block mainly presents the position and velocity information of the vehicle in a high frequency. A GNSS/INS integration algorithm based on Kalman filter is performed in the integration filter to provide the final navigation results and IMU errors. The INS predicts the move Doppler of the vehicle and feeds it to the NCO control algorithm. The adaptive tracking is capable of extracting carrier phase when GNSS signals are in good condition. It can also avoid the impact of loop filter output deterioration on NCO control to ensure the quality of pseudo-range and Doppler. Other than that, the proposed MEMS INS aided weight control algorithm will suppress the deterioration of pseudo-range accuracy caused by signal attenuation and multipath, thereby helping to improve the quality of NCO control information and deep couple positioning accuracy in GNSS-challenged environment.

An antenna receives the GNSS signals and feeds them to a radio frequency(RF) front end in which the GNSS signals are down converted to IF signals, which can be expressed as follows [45,46]:

(1) $$\begin{array}{c} S_{IF,I}\left(t\right)=AC\left(t\right)D\left(t\right)cos\left(2\pi \left(f_{IF}+f_{d}\right)t+\psi_{0}\right)+n_{IF}\left(t\right).\\ S_{IF,Q}\left(t\right)=AC\left(t\right)D\left(t\right)sin\left(2\pi \left(f_{IF}+f_{d}\right)t+\psi_{0}\right)+n_{IF}\left(t\right). \end{array}$$

where *A* is the amplitude of IF signal, C(t) and D(t) are the Pseudo Random Number (PRN) code and navigation data bit respectively. $f_{IF}$ is the IF frequency, $f_{d}$ is the Doppler frequency, and $\psi_{0}$ is the initial phase. We use $n_{IF}\left(t\right)$ as the noise of IF signal which is modeled as zero-mean white Gaussian noise (WGN). The subscripts *I* and *Q* represent the in-phase and quadrant-phase signals respectively.

The IF signals go through a mixer and a correlator, producing the correlated signals. Then an integration and dump operation is performed, the results have the following form:

(2) $$\begin{array}{c} I_{p}\left(t_{1}\right)=AD\left(t_{1}\right)sinc\left(\delta_{f}T_{coh}\right)cos\left(2\pi \delta_{f}\left(t_{1}+\frac{T_{coh}}{2}\right)+\delta_{\psi_{0}}\right).\\ Q_{p}\left(t_{1}\right)=AD\left(t_{1}\right)sinc\left(\delta_{f}T_{coh}\right)sin\left(2\pi \delta_{f}\left(t_{1}+\frac{T_{coh}}{2}\right)+\delta_{\psi_{0}}\right). \end{array}$$

where $T_{coh}$ is the coherent integration time, $\delta_{f}$ is the Doppler estimation error, and $\delta \psi_{0}$ is the initial phase estimation error. Note that the period of the integration can be no longer than a navigation bit because of the bit hopping. The measurement pre-processing operates on the accumulated correlator outputs and then it performs the discriminator function to form Kalman filter measurements.

A MEMS IMU is employed to estimate vehicle movements in the GIDCSR. Its bias and scale-factor errors are included in the functional model of the IMU and are estimated by the GNSS/INS integration filter. The error model of the IMU is expressed as:

(3) $$\begin{array}{c} {\hat{\omega }}_{\mathrm{ib}}^{b}=\left(I+s_{g}\right)\omega_{\mathrm{ib}}^{b}+b_{g}+n_{g}\\ {\hat{f}}^{b}=\left(I+s_{a}\right)f^{b}+b_{a}+n_{a}, \end{array}$$

where ${\hat{\omega }}_{\mathrm{ib}}^{b}$ and ${\hat{f}}^{b}$ are raw output of the IMU. We use $\omega_{\mathrm{ib}}^{b}$ and $f^{b}$ to denote the truth value of the angular velocity and specific force. $b_{g}$ and $b_{a}$ are the biases of the gyroscope and accelerometer respectively. $s_{g}$ and $s_{a}$ are scale-factor errors. n is the noise. In the GNSS/INS integration filter, the state vector has the following form:

(4) $$\begin{array}{c} x\left(t\right)={\left[{\left(\delta r_{INS}^{n}\right)}^{T}\;{\left(\delta v_{INS}^{n}\right)}^{T}\;\phi^{T}\;{b_{g}}^{T}\;{b_{a}}^{T}\;{s_{g}}^{T}\;{s_{a}}^{T}\right]}^{T}, \end{array}$$

where $\delta r_{INS}^{n}$, $\delta v_{INS}^{n}$, ϕ are the position, velocity and attitude error, respectively.

In deeply coupled mode, the INS output is also used for motion Doppler prediction between vehicle and satellites to assist GNSS tracking loops [18]. With the predicted motion Doppler, along side with the receiver clock noise estimation presented by the integration filter, the tracking loops need only to tolerate the INS estimation error, instead of the vehicle dynamic. In other words, the tracking loops can work under a quasi-static situation, which permits us to compress the bandwidth and lengthen the integration time to suppress thermal noise.

In order to improve the performance of the tracking loops in GNSS-challenged urban environments, with the INS assistance, an adaptive open-close loop strategy is applied, which plays a significant role in the GIDCSR. This strategy permits each channel to switch between open-loop and closed-loop tracking adaptively, improving the accuracy and robustness of tracking loop greatly. After a Fast Fourier Transform (FFT) operation on the integrated signals, an FFT-based signal-to-noise ratio (SNR) indicator is calculated. Compared with carrier-to-noise ratio (CNR), the FFT-based SNR is calculated in a higher frequency and is more robust. According to the FFT-based SNR, the receiver determines whether to cut-off the loop filter to NCO switch. If the signals are too weak or the loop loses lock, the switch is cut off and an open-loop tracking of the signal is applied. If all the satellite channels are in closed-loop mode, the GIDCSR is a classic GNSS/INS scalar-based deeply coupled structure [47]. When all the channels are in open-loop mode, the GIDCSR works as a GNSS/INS federated vector-based deeply coupled structure [43]. Therefore, with the proposed adaptive open-close loop strategy, the GIDCSR can change between scalar and vector structure flexibly. When the GNSS signal is strong, the channel in closed-loop mode can track the carrier phase accurately. When the GNSS signal is attenuated, blocked or reflected, the channel in open-loop mode can track the pseudo-range robustly.

In addition, we propose an INS Aided Pseudo-range Weight Control Algorithm (IWA) to eliminate the pseudo-range gross errors caused by attenuated, blocked or reflected GNSS signals. The GNSS/INS integrated navigation position of last epoch is used to estimate the distance between the vehicle and each satellite. Then a reference satellite is chosen and a double-difference alike operation is performed. By this way, the errors of the pseudo-ranges can be estimated and applied to weight the pseudo-ranges. A detailed description will be provided later. It turns out that IWA can improve the quality of the pseudo-ranges and position greatly in GNSS-challenged environments.

### 2.2. Adaptive Open-Close Loop Strategy

In urban environments, GNSS signals are frequently reflected, blocked and weakened. When the signals are too weak (basically with CNR lower than 20 dB-Hz) or blocked, the discriminator outputs cannot truly reflect the difference between NCOs and input signals, caused to incorrectly control NCOs. A common strategy in a pure GNSS receiver is to announce the tracking loop loses lock and a reacquisition is performed. This way of tracking recovery is neither fast nor accurate, and it will be nearly useless when the signals are blocked frequently, which is very common in urban environments.

**Figure 2:**
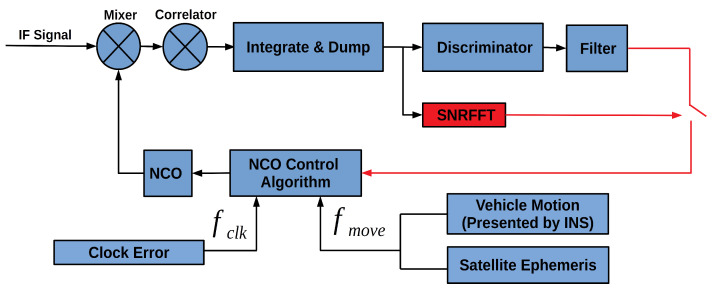
Open-close loop strategy in GNSS/INS Deeply-coupled Software-defined Receiver (GIDCSR).

This paper presents an open-close loop strategy to overcome the problem, shown in Figure 2. With the vehicle position presented by INS and the satellite position, the line-of-sight unit vector of the vehicle relative to the satellite is calculated in the first place. Then the line-of-sight motion Doppler $f_{move}$ is calculated according to the vehicle velocity and satellite velocity. Every time the Kalman filter is updated, the frequency deviation of the receiver clock $f_{clk}$ is estimated. When SNRFFT indicates strong signal strength, the Doppler error output by the local filter, the motion Doppler and receiver clock deviation jointly control the NCO in which the carrier phase can be extracted. When the SNRFFT results present unacceptable signal noise, the loop filter output is not sent to control the NCO, and only the motion Doppler and clock deviation are used to control the NCO, turning the tracking loop into an open-loop status. Three types of NCO control information can be updated in different frequency.

The strategy can improve the continuity of the signal tracking greatly. When part of the satellites are blocked, the INS errors can still be corrected by visible satellite, which means the aiding Doppler errors provided by INS will not diverge. Therefore, with the assistance of INS aiding information, the NCO error would not increase quickly. When the blocked satellites are available again, the tracking loop can recover accurate carrier phase tracking rapidly. In other words, the strong signal channels assist those channels with weak signals, which meets the philosophy of vector based tracking. This strategy combines the advantages of both scalar and vector based structures. When a channel is in closed loop, it can track carrier phase steadily. And when the signals are weak and frequently blocked, the open loop tracking can improve the robustness of tracking.

**Figure 3:**
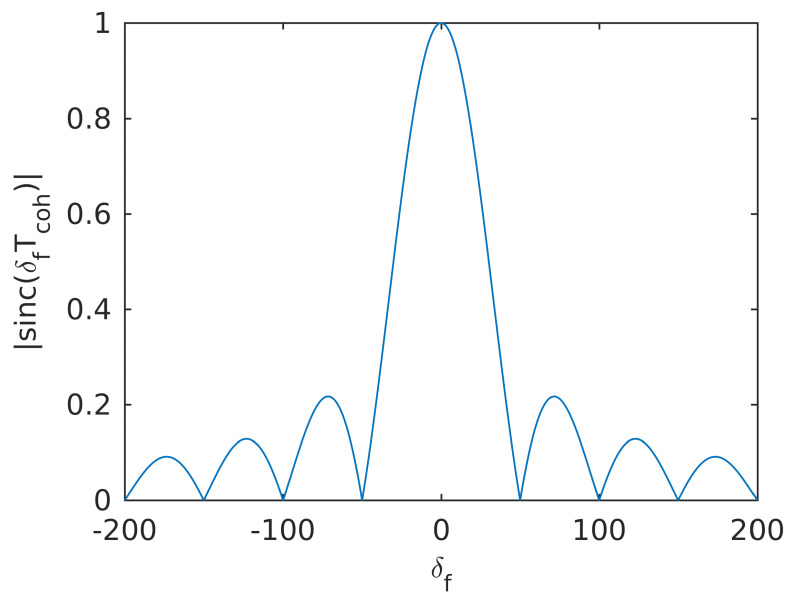
Signal amplitude with respect to Doppler error (with 20 ms of coherent integration).

The traditional approach to estimate the power of GNSS signals is called the Narrowband-Wideband Power Ratio (NWPR) [48]. NWPR calculates the ratio between wideband power (WBP) with bandwidth $1/T_{coh}$ and the narrowband power (NBP) with bandwidth $1/MT_{coh}$. Typically the $T_{coh}$ is set to 1 ms and *M* is set to 20. This approach is sensitive to the Doppler estimation error because 20 ms integration time is used. When the coherent integration time is set to 20 ms, the relationship between the amplitude of the integrated signal and the Doppler error is shown in Figure 3. The amplitude of the integrated signal drops to zero when the Doppler error is 50 Hz. We propose a faster and more robust FFT-based indicator is presented to outcome the shortcomings of NWPR.

**Figure 4:**
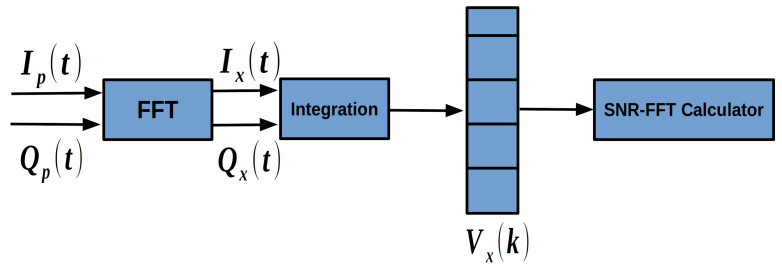
FFT-SNR calculation.

The scheme of SNR-FFT is shown in Figure 4. SNR-FFT takes correlator integration results of GNSS signals $I_{p}\left(t\right)$ and $Q_{p}\left(t\right)$ as input. The complex form of (2) is as follows:

(5) $$\begin{array}{cc} R_{p}\left(t\right) & =I_{p}\left(t\right)+jQ_{p}\left(t\right)\\ \; & =AD\left(t\right)sinc\left(\delta_{f}T_{coh}\right)exp\left(j\left(2\pi \delta_{f}\left(t+\frac{T_{coh}}{2}\right)+\delta_{\psi_{0}}\right)\right). \end{array}$$

The discrete-time model of the accumulated signal over the *i*th integration interval can be expressed as:

(6) $$\begin{array}{c} I_{p}\left(i\right)=\frac{1}{N_{coh}}\sum_{n=1}^{N_{coh}}i_{p}\left(iN_{coh}+n\right)\\ Q_{p}\left(i\right)=\frac{1}{N_{coh}}\sum_{n=1}^{N_{coh}}q_{p}\left(iN_{coh}+n\right), \end{array}$$

where $i_{p}\left(i\right)$ and $q_{p}\left(i\right)$ are the outputs of correlator and $N_{coh}$ epochs of data are accumulated.

The FFT operation can be expressed as:

(7) $$v_{x}\left(k\right)=\sum_{i=1}^{N}R_{p}\left(i\right)exp\frac{-2\pi j(i-1)(k-1)}{N},$$

where $R_{p}\left(i\right)=I_{p}\left(i\right)+jQ_{p}\left(i\right)$ is the time domain input signal, $v_{x}\left(k\right)$ is the frequency domain output signal and the subscript *x* means the signal is converted to frequency domain. N is the number of FFT processing points.

**Figure 5:**
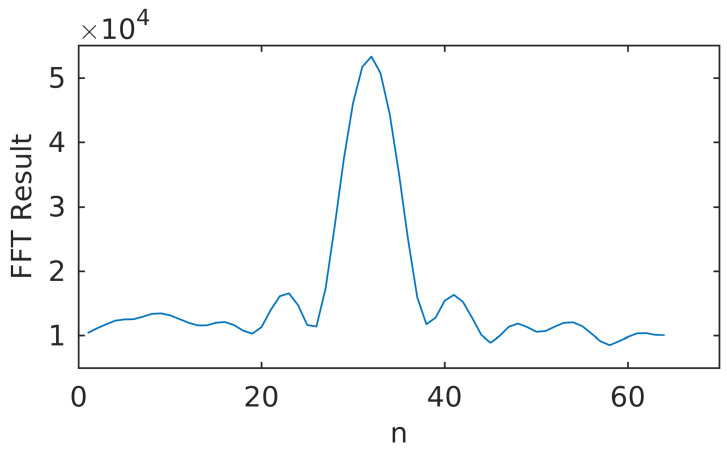
Non-coherent FFT result.

The FFT result reflects the power density distribution with respect to the Doppler frequency residual. As is shown in (5), the integrated signal is a single tone complex signal. Although the signal is flooded in the noise in time domain, the power of the single tone complex signal can stand out at the specific frequency point in frequency domain. Note that the FFT performs a series of multiplication and addition operations. Essentially the FFT method is equivalent to coherent integration. So the length of the input of the signals can be no longer than 20 ms because of the bit-flipping. The resolution of the FFT method $f_{r}$ can be denoted as:

(8) $$f_{r}=\frac{f_{s}}{N_{s}},$$

where $f_{s}$ is the sampling rate and $N_{s}$ is the number of FFT processing points. Sometimes the signals can be weak and the peak of the FFT results is hard to detect, making it difficult to calculate the SNR-FFT indicator. To solve the problem, an accumulation function is performed on the FFT results:

(9) $$V_{x}\left(k\right)=\sum_{m=1}^{N_{nocoh}}v_{x}^{\left(m\right)}\left(k\right),$$

where $N_{nocoh}$ FFT results are accumulated and $v_{x}^{\left(m\right)}\left(k\right)$ is the *m*th FFT result in the accumulation interval with index *k*. In this way the power of the signals are accumulated. The integrated FFT result is shown in Figure 5. The peak is where the Doppler frequency residual stands. The rest is noise. We can see that the peak is not in a single frequency point. It is because that the resolution of the FFT method is not infinite. The power of the specific frequency point is spread to the several points near it. Let $E_{1}=max\left(V_{x}\left(k\right)\right)$ denote the maximum energy, $E_{2}$ and $E_{3}$ denote the maximum energy adjacent to $E_{1}$. An FFT based SNR indicator can be calculated as:

(10) $${\mathrm{SNR}}_{\mathrm{FFT}}=\frac{(E_{1}+E_{2}+E_{3})/3}{\left(E_{A}-E_{1}-E_{2}-E_{3}\right)/\left(N-3\right)},$$

where $E_{A}$ is the total energy of signal and noise in the FFT bandwidth:

(11) $$E_{A}=\sum_{i=1}^{N}V_{x}\left(k\right).$$

In (10), $(E_{1}+E_{2}+E_{3})/3$ denotes the power of signal and $\left(E_{A}-E_{1}-E_{2}-E_{3}\right)/\left(N-3\right)$ is the estimation of power of noise. The FFT based SNR can be calculated this way. SNR-FFT can detect very weak signals. Also it can reflect the change of satellite signal strength in a timely manner, so that the loop strategy can make corresponding adjustments in time. In GIDCSR, when the SNR-FFT detects a signal with $C/N_{0}$ lower than 25 dB-Hz, which is set as the carrier phase tracking threshold, the open-loop tracking mode is turned on.

### 2.3. INS Aided Pseudo-Range Weight Control Algorithm

The common approaches to weight GNSS pseudo-range are based on signal $C/N_{0}$ and satellite elevation, which are reasonable in open sky environments. In urban environments, these approaches are not suitable because of the existence of not line of sight (NLOS) signals and severe multipath [49]. As a solution with both high level of accuracy and robustness in GNSS-challenged environments, the INS aided pseudo-range weight control algorithm is proposed.

The pseudo-range of the satellite with PRN i can be denoted as:

(12) $$\rho^{\left(i\right)}=r+c\left(\phi t_{u}-\phi t^{\left(i\right)}\right)+cI+cT+\epsilon_{\rho },$$

where, $\rho^{\left(i\right)}$ is the pseudo-range observation, $r+c\left(\phi t_{u}-\phi t^{\left(i\right)}\right)+cI+cT+\epsilon_{\rho }$ is the predicted pseudo-range. If the predicted pseudo-range could be calculated accurately, it can be applied to estimate the gross errors of pseudo-range observation. *r* is the absolute distance of Line of Sight (LOS) between the *i*th satellite and the receiver. $\phi t_{u}$ and $\phi t^{\left(i\right)}$ denote the receiver clock error and satellite clock error respectively. *I* and *T* represent the ionosphere and the troposphere propagation errors respectively. Considering each satellite is equipped with an atomic clock, the impact of $\phi t^{\left(i\right)}$ can be ignored. In a short time, the ionosphere and troposphere propagation errors do not vary severely. As a result, the unstable factors which can affect the quality of pseudo-range prediction are the receiver clock error $\phi t_{u}$ and the absolute distance *r*. The absolute distance depends on the satellite position and the vehicle position. We can calculate the satellite position accurately with ephemeris. So the accuracy of pseudo-range is mainly affected by the receiver clock error and the vehicle position. In other words, if the receiver clock error and absolute distance items are removed from (12), we can get a slowly varying curve. The slowly varying character can be used to eliminate the gross errors of pseudo-range and to weight pseudo-range. The details are given as follows.

**Figure 6:**
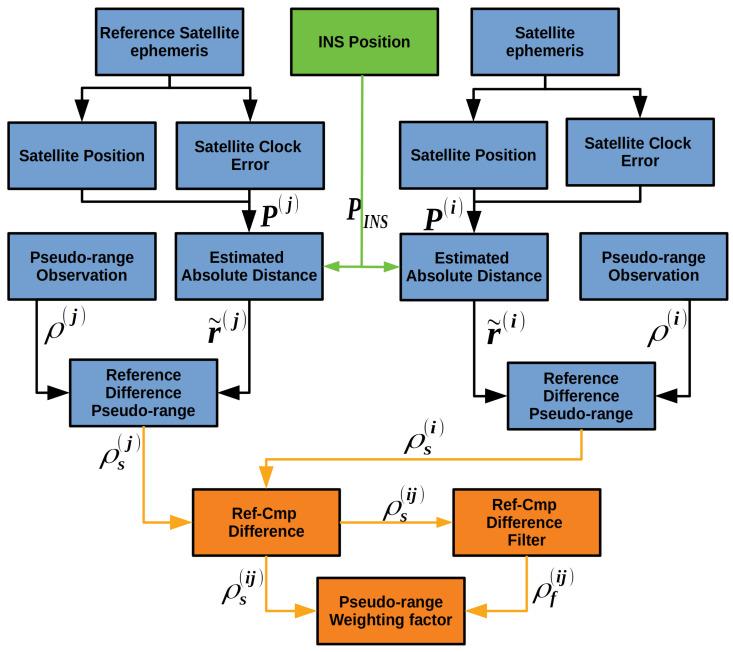
Diagram of INS aided pseudo-range weight control algorithm in GIDCSR.

The flow diagram of IWA is shown in Figure 6. Firstly a reference satellite is chosen from all the tracking satellites with the principle that the chosen satellite has the highest elevation angle and the strongest $C/N_{0}$. The positions and clock errors of the reference satellite and all other satellites are obtained by the ephemeris. Since the accuracy of INS is high in a short time and is not affected by environments, it can provide the vehicle position. After the compensation of lever arm, the position coordinate in Earth Centered Earth Fixed (ECEF) frame is denoted as $P_{\mathrm{INS}}\left(x_{INS},y_{INS},z_{INS}\right)$. With the already known satellite position coordinate $P^{\left(i\right)}\left(x^{\left(i\right)},y^{\left(i\right)},z^{\left(i\right)}\right)$, the estimation of the absolute distance between the satellite and the vehicle can be calculated:

(13) $${\tilde{r}}^{\left(i\right)}=\sqrt{{\left(x^{\left(i\right)}-x_{INS}\right)}^{2}+{\left(y^{\left(i\right)}-y_{INS}\right)}^{2}+{\left(z^{\left(i\right)}-z_{INS}\right)}^{2}}.$$

Then the absolute distance is deducted from pseudo-range observations:

(14) $$\begin{array}{cc} \rho_{s}^{\left(i\right)} & =\rho^{\left(i\right)}-{\tilde{r}}^{\left(i\right)}\\ \; & =r_{e}+c\left(\phi t_{u}-\phi t^{\left(i\right)}\right)+cI+cT+\epsilon_{\rho }, \end{array}$$

where $r_{e}$ is the estimation error of absolute distance. The left items in (14) vary slowly except the receiver clock error. Next step is to eliminate the receiver clock error, which leads a difference operation between reference satellite and other satellite:

(15) $$\rho_{s}^{\left(ij\right)}=\rho_{s}^{\left(i\right)}-\rho_{s}^{\left(j\right)}.$$

In this way, the receiver clock error is eliminated and the remain items are the ionosphere and troposphere propagation errors, which vary very slowly. The slowly varying items are then fed to a smooth filter. An α smoother will do the work.

(16) $$\rho_{f}^{\left(ij\right)}\left(n\right)=\alpha \rho_{s}^{\left(ij\right)}\left(n\right)+\left(1-\alpha \right)\rho_{s}^{\left(ij\right)}\left(n-1\right).$$

Finally the difference between $\rho_{f}^{\left(ij\right)}$ and $\rho_{s}^{\left(ij\right)}$ is calculated:

(17) $$\rho_{Df}^{\left(ij\right)}=\rho_{s}^{\left(ij\right)}-\rho_{f}^{\left(ij\right)}.$$

**Figure 7:**
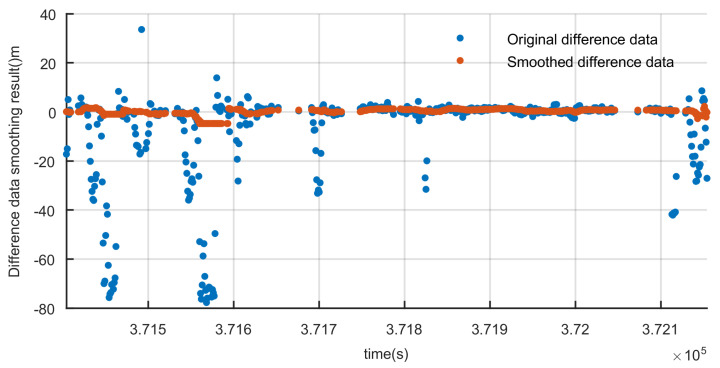
Difference data smoothing result.

In urban environments, if a gross error is contained in the pseudo-range observation, this error can be easily detected in this way. The $\rho_{s}^{\left(ij\right)}$ and $\rho_{f}^{\left(ij\right)}$ of a GPS satellite with PRN 5 are plotted Figure 7. Based on the fact that under vehicle dynamics, the ionosphere and troposphere propagation errors vary slowly, the differences between the original and smoothed difference data results can reflect the errors of the pseudo-ranges. And these errors are usually caused by the signal’s blocking and reflection.

**Figure 8:**
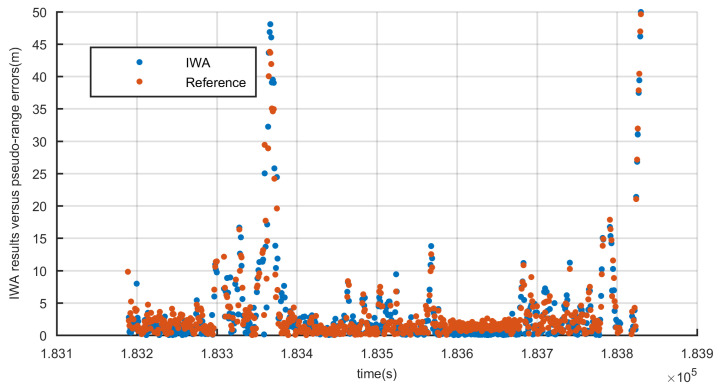
IWA results versus true pseudo-range errors.

To prove above point, the IWA results $\rho_{Df}^{\left(ij\right)}$ and pseudo-range errors are plotted in Figure 8. The pseudo-range error is the difference between pseudo-range observation and reference pseudo-range. According to the high precision vehicle position provided by reference system and the position of satellite, reference pseudo-range can be calculated. The deduction of reference pseudo-range will be described in detail in Section 4. In Figure 8, IWA results and pseudo-range errors show high consistency. Therefore, the IWA results can correctly reflect the pseudo-range gross errors and be used to weight the pseudo-ranges, which will eliminate most of the gross errors and improve the accuracy of position greatly. In practice, the three stages weighting algorithm is applied:

(18) $$w_{i}=\left\{\begin{array}{c} 1\;\;\;\;\;\;\left|\rho_{DF}^{\left(ij\right)}\right|<=K_{ths0}\\ \frac{K_{ths1}-\left|\rho_{DF}^{\left(ij\right)}\right|}{K_{ths1}-K_{ths0}}\;\;K_{ths0}<\left|\rho_{DF}^{\left(ij\right)}\right|<=K_{ths1}\\ 0\;\;\;\;\;\;\left|\rho_{DF}^{\left(ij\right)}\right|>K_{ths1}, \end{array}\right.$$

where $w_{i}$ is the weighting value of the $i_{th}$ satellite pseudo-range observation. We set two threshold values $K_{ths0}$ and $K_{ths1}$. When the estimated noise is smaller than $K_{ths0}$, the pseudo-range is weighted as 1. When the estimated noise is unbearable, the pseudo-range is simply thrown away. Not only can IWA improve the quality of pseudo-range, but also it can improve the accuracy of position results by weighting the pseudo-range.

## 3. Experiment Setup

In order to assess the performance of GIDCSR, a field vehicle test was performed in the deep urban area of Wuhan China.

**Figure 9:**
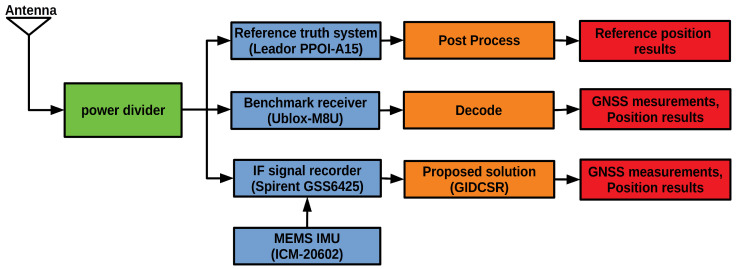
Scheme of experiment.

**Figure 10:**
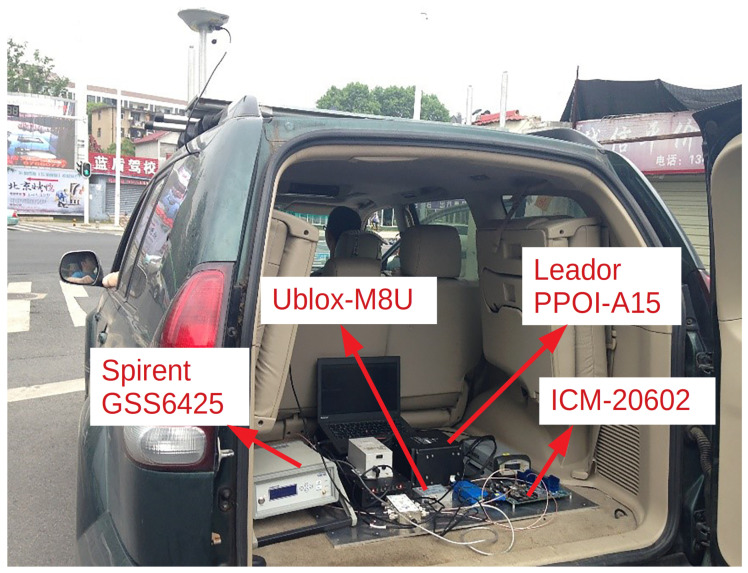
Testing vehicle and equipment.

As is shown in Figure 9, the reference system is a high precision Position and Orientation system (POS)—Leador PPOI-A15 [50], which is composed of a surveying GNSS receiver and a ring laser gyro (RLG) IMU. Leador PPOI-A15 is capable of providing centimeter level positioning results by post processing, which meets the requirement of a reference for the experiment. The RF signals are recorded and downconverted to IF by a Spirent GSS6425 multiple constellation record playback system [51]. The raw data of a low-cost MEMS IMU ICM-20602 is recorded synchronously. IF signals and ICM-20602 data are fed to GIDCSR for processing. Meanwhile, an M8U evaluation kit from u-blox is installed. u-blox is a leading company in the area of vehicle navigation. M8U is a typical GNSS/INS tightly-coupled navigation product with a low cost MEMS IMU. It can provide excellent navigation results for vehicles in urban environments. The observations and position results of M8U will be compared with the results of our GIDCSR. Since the specification of the IMU inside M8U is not provided by the datasheet. In order to ensure the fairness of comparison, we performed an Allan variance (AVAR) analysis based on the two IMUs’ long time static data. The result is shown in Table 1. The error of the two IMUs is basically at the same line. The test equipment installation on the vehicle is shown in Figure 10.

**Figure 11:**
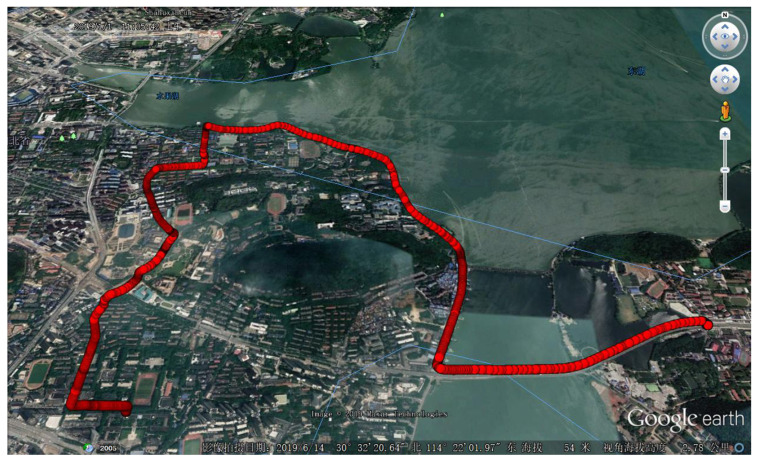
Test trajectory in urban area.

The experiment data was collected near Wuhan University. The vehicle trajectory is shown in Figure 11.

**Figure 12:**
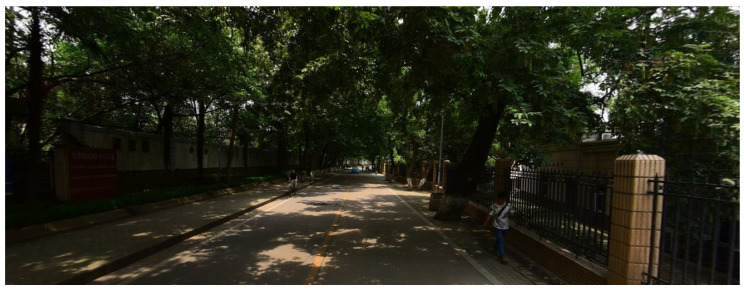
Test scene under the shade of trees.

**Figure 13:**
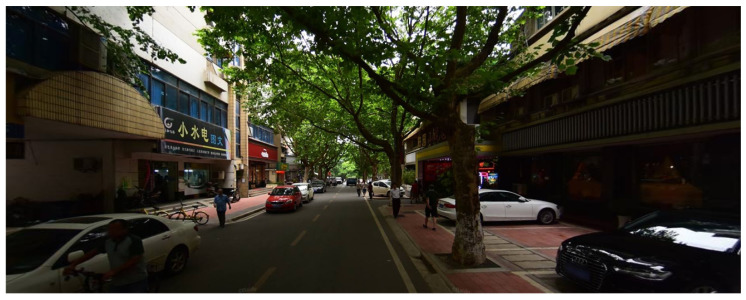
Test scene beside buildings.

The vehicle went across a road under a shade of trees near a lake first, where the GNSS signals are weakened and reflected (Figure 12). Then a narrow road (Figure 13) with buildings and trees on both sides of it passed, where the signals were weakened and some satellites were frequently blocked. About 30 min of data was collected. Based on the collected data, the performance of deep couple before and after applying the two proposed techniques were analyzed, and then the performance comparison between GIDCSR and u-blox M8U was made in pseudo-range, GNSS position and GNSS/INS integrated position.

## 4. Experiment Results and Analysis

GIDCSR is developed on basis of non-coherent deep couple structure. Two key techniques (adaptive open-close tracking loops and IWA) are proposed to improve the continuity of tracking and accuracy of pseudo-range and Doppler in GNSS-challenged urban environments. In this section the performance analysis of GIDCSR is performed, and the comparison between GIDCSR and typical non-coherent deep couple is made. As two comprehensive navigation solutions facing the GNSS-challenged environments, GIDCSR and M8U’s performance is compared. Since the GNSS signals are terribly weakend and affected by multipath in deep urban areas, the coherent deep couple which requires continous accurate carrier phase tracking can not work in such environments. So the comparison between GIDCSR and coherent deep couple is not made. The detailed comparison of different deep couple structures based on simulation data can be found in [52].

### 4.1. Deep Couple Performance Analysis

This section presents a performance analysis to assess the contribution of the adaptive open-close tracking loops and INS aided pseudo-range weight control algorithm in GNSS-challenged urban environment. First of all, the performance of adaptive open-close tracking loops is assessed. Then, the contribution of INS aided pseudo-range weight control algorithm is analyzed. Finally, the positioning performance before and after applying the two algorithms is performed.

#### 4.1.1. Adaptive Open-Close Tracking Loops Analysis

In this part the pseudo-range quality of GIDCSR before and after disabling the adaptive open-close tracking strategy is compared. The INS aided pseudo-range weight control algorithm is disabled to eliminate the influnce of INS.

To compare the error of pseudo-range, a reasonable pseudo-range error analysis method is developed firstly. The reference POS system can provide accurate position of the receiver (denoted as $P_{\mathrm{Ref}}\left(x_{Ref},y_{Ref},z_{Ref}\right)$) and the positions of all visible satellites can be provided by the ephemeris. With the already known positions of the receiver and each visible satellite, the geometrical distances between the receiver and satellites are calculated:

(19) $$r_{g}^{\left(i\right)}=\sqrt{{\left(x^{\left(i\right)}-x_{Ref}\right)}^{2}+{\left(y^{\left(i\right)}-y_{Ref}\right)}^{2}+{\left(z^{\left(i\right)}-z_{Ref}\right)}^{2}}.$$

Next the geometrical distance is deducted from the pseudo-range observation. Then a reference satellite *k* is chosen, the difference between satellite *k* and other satellites are calculated, which contains the slowly-varying ionosphere and troposphere errors:

(20) $$\delta_{i}=\left(\rho^{\left(i\right)}-r_{g}^{\left(i\right)}\right)-\left(\rho^{\left(k\right)}-r_{g}^{\left(k\right)}\right).$$

We can observe the pseudo-range errors easily through $\delta_{i}$. It is worth noting that the pseudo-range error analysis method and IWA algorithm have similar ideas. Both of them performs a double difference alike operation. The former uses the high-precision positions provided by the reference system to assess the quality of pseudo-ranges, while the latter is to detect the gross errors of the pseudo-ranges to produce better weights.

In our analysis, GPS satellite 7 and BD satellite 7 were selected as reference satellites, for the observation conditions of them were good throughout the whole test.

**Figure 14:**
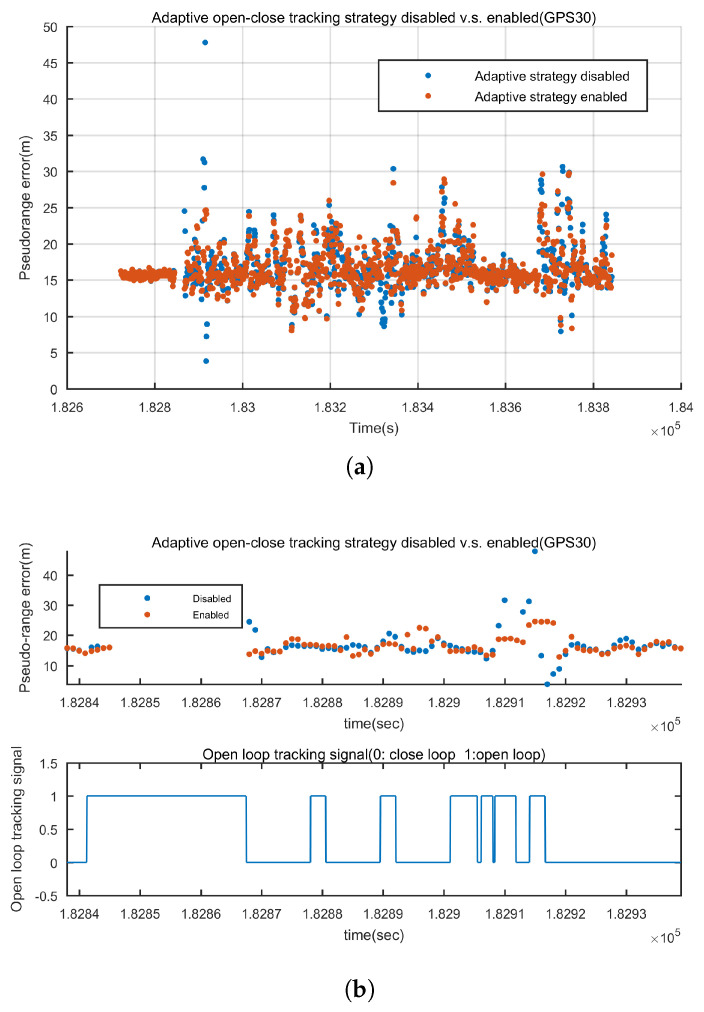
Pseudo-range error before and after applying the adaptive open-close loop tracking strategy of GPS 30 (**a**) Pseudo-range error during the whole experiment (**b**) From 182,840 s to 182,930 s, GNSS signals are frequently blocked and the adaptive open-close loops presented better pseudo-ranges.

**Figure 15:**
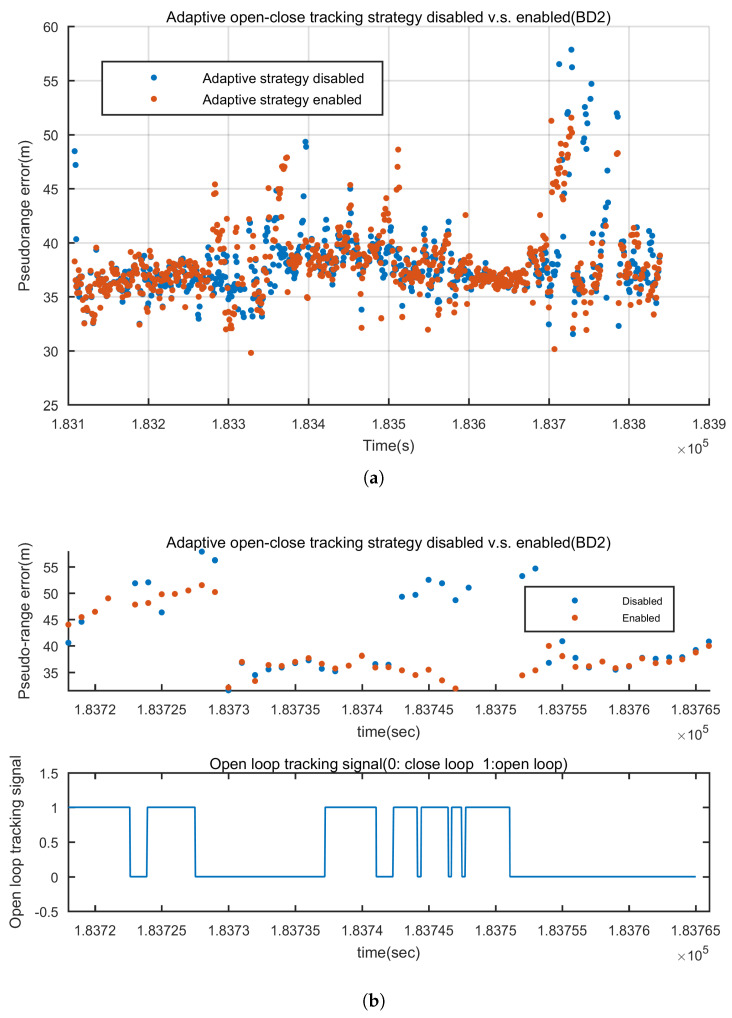
Pseudo-range error before and after applying the adaptive open-close loop tracking strategy of BD 2 (**a**) Pseudo-range error during the whole experiment (**b**) From 183,720 s to 183,760 s, GNSS signals are frequently blocked and the adaptive open-close loops presented better pseudo-ranges.

For instance, the pseudo-range errors of GPS 30 and BD 2 before and after disabling the adaptive open-close loops are shown in Figure 14 and Figure 15. Figure 14a is the error of the pseudo-range throughout the whole experiment. It is shown that after disabling the adaptive open-close loops, the gross errors occur more frequently. When the GNSS signals are blocked for a short time, the adaptive open-close loops is capable of maintaining the tracking and presenting more accurate pseudo-range. To demontrate the point, the local zooms are shown in Figure 14b. Also the open loop tracking sign is shown. From 182,840 s to 182,870 s, GPS 30 was blocked for about 30 s. It can be clearly seen that the adaptive open-close loop performed 30 s of open loop tracking. It presented 2 more epochs of pseudo-range with satisfying accuracy and when the signal was available again, it recovered tracking rapidly and presented better pseudo-range. We can observe the same pattern from 182,910 s to 182,920 s. BD 2 shows the same result. In GNSSS-challenged environments, adaptive open-close loops can improve the continuity of tracking and the accuracy of pseudo-range.

#### 4.1.2. INS Aided Pseudo-Range Weight Control Algorithm Analysis

**Figure 16:**
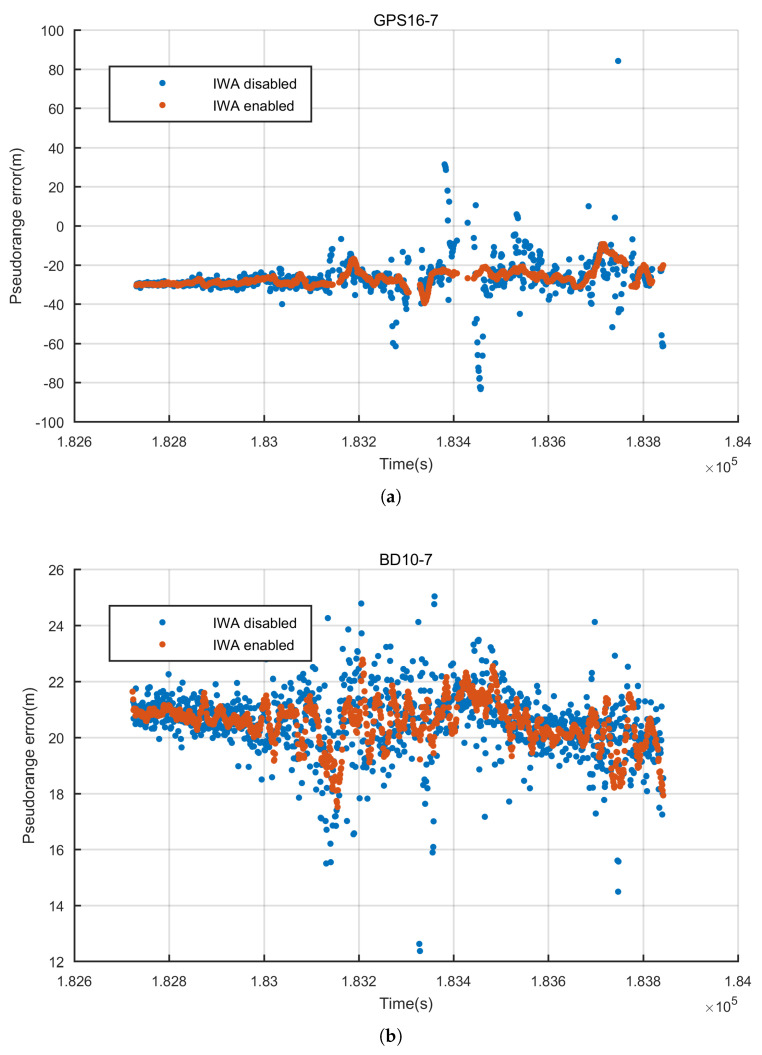
Pseudo-range error before and after applying INS aided weight control algorithm (**a**) Pseudo-range error comparison of GPS16 (**b**) Pseudo-range error comparison of BD10.

The contribution of the INS aided pseudo-range weight control algorithm is analyzed in this section. The pseudo-range errors are compared before and after disabling IWA, in which the adaptive open-close tracking loops are enabled. For instance, the results of two satellites—GPS 16 and BD 10, are plotted (Figure 16) and eight satellites’ statistical indicators are calculated (Table 2). The results indicate that IWA can improve the quality of pseudo-range greatly. The gross errors of pseudo-range are detected and compensated. The accuracy of pseudo-range is improved by at least 9%.

#### 4.1.3. Deep Couple Positioning Analysis

**Figure 17:**
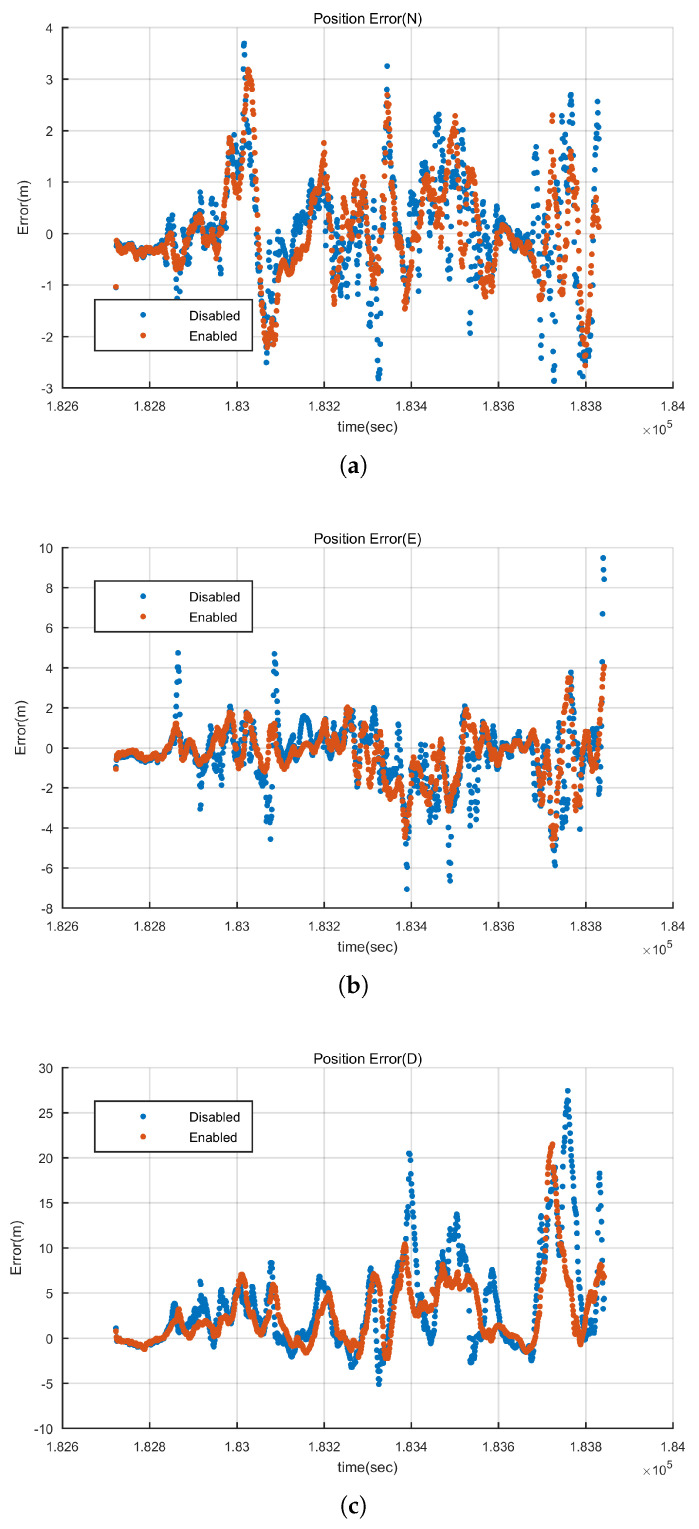
Integration errors comparison before and after applying adaptive tracking loops and INS aided wight control algorithm. (**a**) North direction (**b**) East direction (**c**) Height.

As described in Section 2, GIDCSR degenerates into typical non-coherent deep couple structure if the adaptive open-close tracking loops and INS aided pseudo-range weight control algorithm are disabled. In order to assess the comprehensive performance of these two algorithms, the integrated positioning results before and after disabling them are compared and analyzed. The results are shown in Figure 17. Take eastern direction as an example, if the two algorithms are disabled, the positioning error increases a lot over the interval from 182,800 s to 183,200 s because of the degradation of GNSS signals. The positioning error increases by two to three times when the GNSS signals degrades. With the help of the adaptive tracking loops and IWA, when the GNSS signal strength is strong, carrier phase can be tracked stably, and if the signal strength degrades in urban environment, the quality of pseudo-range and Doppler is ensured. The harsher the scenario is, the more obvious the effect of the two algorithms will be.

### 4.2. Experiment Results Comparison Between GIDCSR and M8U

The test results of the M8U and GIDCSR are analyzed in three steps. We compare the errors of pseudo-range observations directly in the first place. Then a common GNSS single-point positioning(SPP) algorithm is applied to process the pseudo-ranges from the two receivers, and the corresponding position errors are compared. Finally, the ultimate position results generated by GNSS/INS couple are compared.

#### 4.2.1. Pseudo-Range Error Analysis

**Figure 18:**
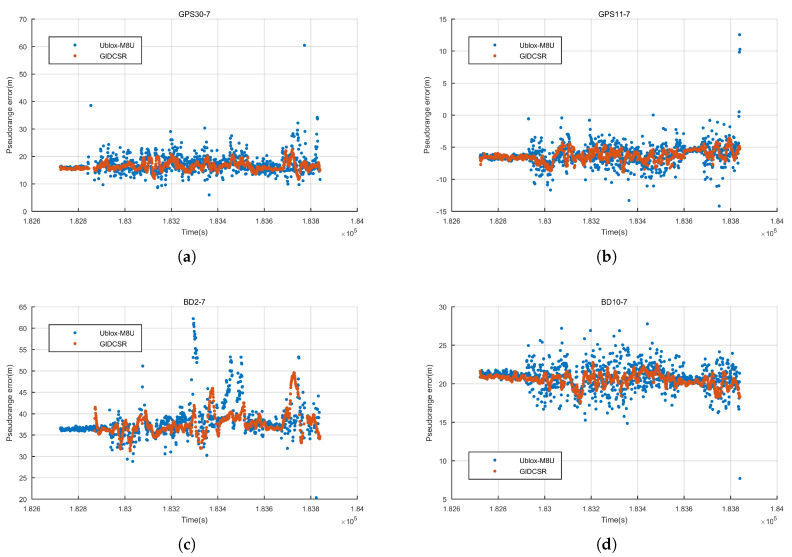
Pseudo-range errors (m) of some satellites. (**a**) GPS satellite 30 with low elevation (**b**) GPS satellite 11 with high elevation (**c**) BD satellite 2 (GEO) (**d**) BD satellite 10 (IGSO).

The pseudo-range errors of two GPS satellites 30,11 and two BD satellite 2,10 were shown in Figure 18 as illustrations. It can be seen that these curves are not near 0 because of the existence of ionosphere and troposphere errors. GPS satellite 30 and 11 are satellites with low and high elevation respectively. Regarding BD constellation, the performance of a GEO satellite 2 and an IGSO satellite 10 is assessed.

During the first 2–3 min, the vehicle ran under open sky. u-blox M8U and GIDCSR both presented equally satisfying pseudo-range quality. The errors of the pseudo-range in open sky were less than 1 m.

After a right turn, the environment became complex. The errors of GIDCSR are much smoother than that of u-blox M8U. On one hand, the proposed open-close loop strategy can improve the continuity and accuracy of the observations. On the other hand, the IWA also plays an important role. Most of the gross errors were detected and compensated, which guaranteed that the pseudo-range errors could be maintained in a relatively low level.

**Figure 19:**
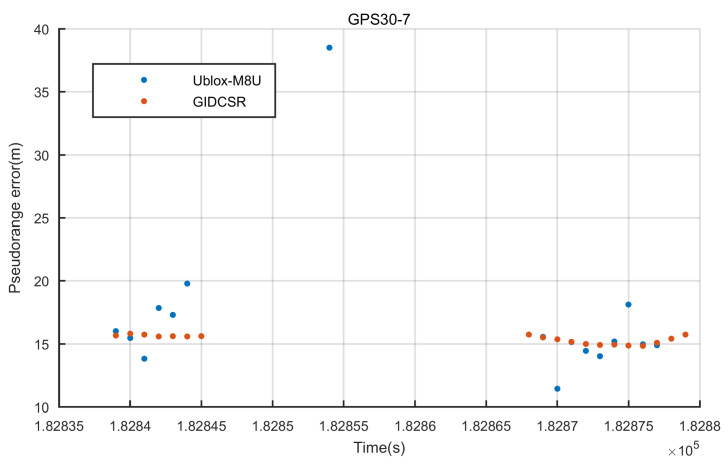
GPS 30 tracking recovery.

GPS satellite 30 was invisible for a while. The pseudo-range error figure was zoomed in the duration (Figure 19). The open-close loop strategy described in Section 2 performed very well in the situation that the satellite was blocked in a short period. Before the satellite turned invisible near 182,845 s, the GIDCSR presented one more epoch of observation than M8U. And the recovery in output of GIDCSR is one epoch faster than that of M8U (near 182,865 s). M8U indeed gave an output of observation in epoch 182,854 s, but the error was too big to provide a satisfying positioning result. When the satellite was blocked, an open loop tracking was conducted. The Doppler provided by IMU could maintain the observation accuracy in a short period. When the satellite turned visible again, the tracking loop could recover carrier phase tracking status rapidly and accurately.

Three indicators of the pseudo-range errors were analyzed—volatility, maximum error and continuity. We calculated the standard deviation (SD), maximum value and proportion of valid epochs of the pseudo-range errors to indicate the quality of the pseudo-range observations (Table 3 and Table 4).

From the tables it can be seen that the noise of each satellite tracked by GIDCSR is much lower than those of M8U. Statistically, the pseudo-range error of GIDCSR is 25.3% to 63.0% smaller than that of M8U. In terms of continuity, GIDCSR outperformed M8U as well. The superiority of GIDCSR’s continuity performance shows up when the elevation angle of the tracking satellite is low. Take GPS as an example, most of the satellites’ proportions of valid epochs of the two racing receivers are almost the same. But GPS satellite 9 and 16 are exceptions. The elevation angle of GPS satellite 9 is around $20^{\circ }$ and that of GPS satellite 16 is around $15^{\circ }$. The two satellites were frequently blocked by roadside buildings, which had negative effect on the tracking loop. As a result, the pseudo-range noise of the two satellites is much larger than those of the other satellites and the continuity gets worse. In this case, GIDCSR’s pseudo-range continuity performance of GPS satellite 9 is 15.11% better than u-blox M8U’s. The corresponding number of GPS satellite 16 is 8.67%. Therefore, from a statistical point of view, the advantages of the open-close loop strategy and IWA in the GIDCSR are confirmed. The open-close loop strategy can improve the continuity and accuracy of signal tracking. And the IWA is capable of lowing the errors of pseudo-range observations.

#### 4.2.2. GNSS Positioning Analysis

GIDCSR is capable of presenting both GNSS and GNSS/INS integration positioning results. However, u-blox M8U’s real time positioning solution is produced by a proprietary GNSS/INS tightly-coupled algorithm according to [53]. In order to compare the quality of the GNSS positioning results of the two GNSS receivers, the RTKLib based PVT module of GIDCSR was applied. GIDCSR is built to a modular design. Thus the PVT module can be applied to process the original pseudo-range observations of M8U.

**Figure 20:**
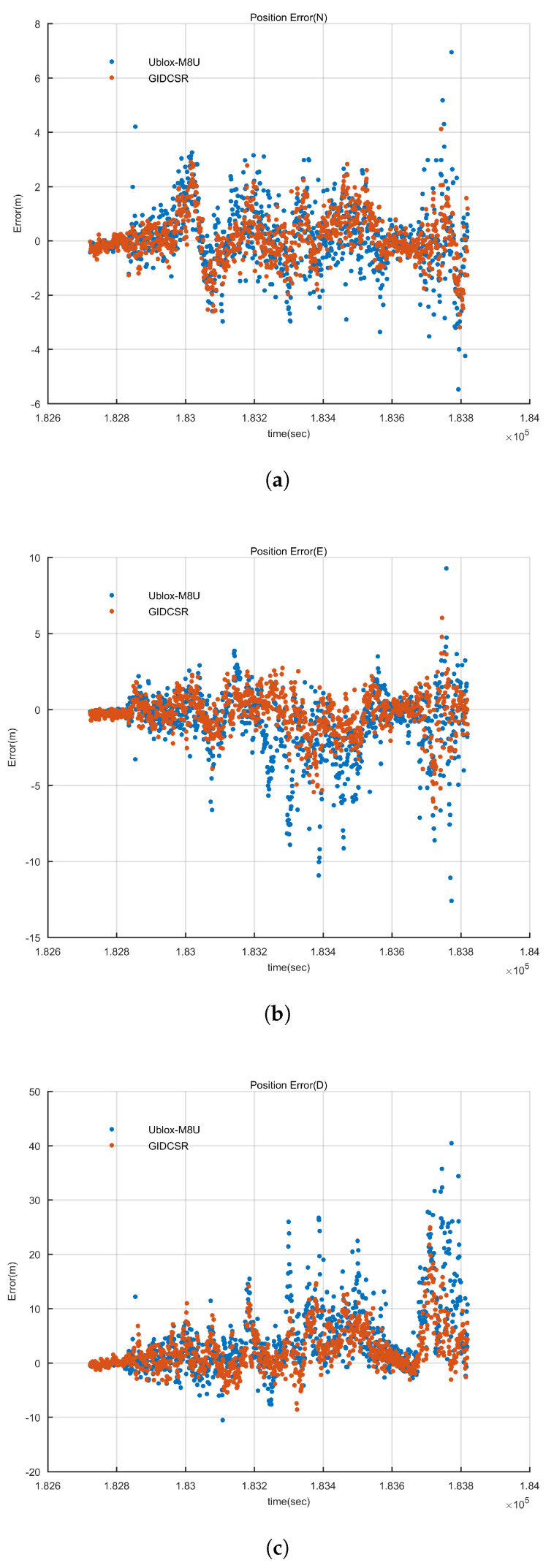
GNSS positioning errors comparison. (**a**) North direction (**b**) East direction (**c**) Height.

The positioning errors in local navigation frame (defined as north, east and down) are shown in Figure 20 and Table 5. GIDCSR outperformed M8U in all three directions. Again, when the GNSS signals are of high quality (during the first few minutes of the experiment), both receivers can provide equally satisfying positioning accuracy. However, when the vehicle is in various complex environments, including shades of trees, roadside buildings and so on, the superiority of GIDCSR shows up. Statistically, GIDCSR’s positioning accuracy is at least 30% better than M8U’s. It can be seen that from 183,200s to 183,600s, the positioning errors increase due to the GNSS signal degradation. During the period GIDCSR can still maintain its positioning accuracy, but the M8U’s positioning errors increase a lot. Positioning results based on pseudo-ranges of the two receivers indicate that GIDCSR can generate better observations than M8U in urban environments again.

#### 4.2.3. GNSS/INS Integration Result Analysis

Through the comprehensive perspective, the comparison of the GNSS/INS integration results of the GIDCSR and u-blox M8U is made. As described before, M8U applies a GNSS/INS tightly coupled algorithm which integrates the GNSS and IMU data in the observation level, while GIDCSR’s integration algorithm is GNSS/INS deep couple which integrates GNSS signals and IMU data in the signal processing level.

**Figure 21:**
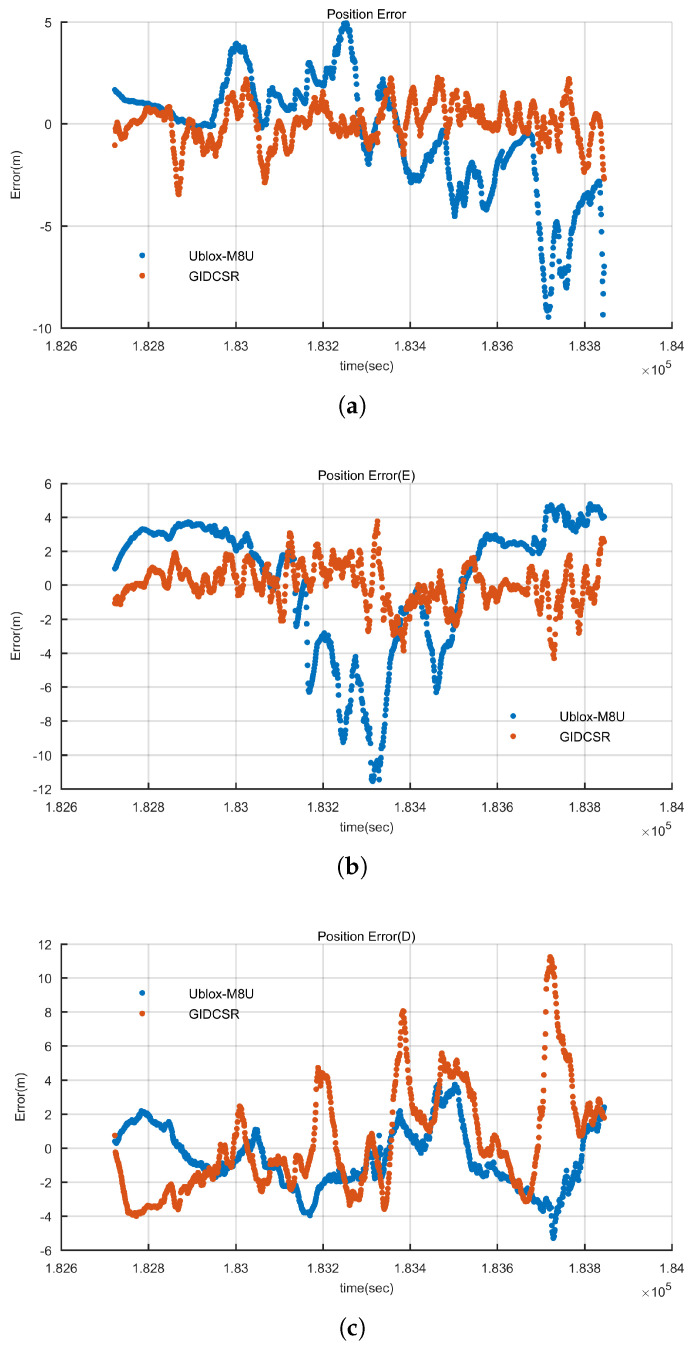
GNSS/INS integration errors comparison. (**a**) North direction (**b**) East direction (**c**) Height.

Similar with the previous subsection, the errors of GNSS/INS integration results of both receivers are shown in Figure 21 and Table 6. When comparing the final positioning errors of GIDCSR and M8U, a completely different pattern shows up.

It can be seen that GIDCSR’s horizontal errors are clearly smaller than M8U’s, which reflects the superiority of GIDCSR. While the SD of GIDCSR’s horizontal error is about 1.5 m, the error of M8U is about 4.8 m. In other words, GIDCSR’s horizontal positioning accuracy is 69% better than M8U’s. Meanwhile, the maxim horizontal error of M8U is also much larger than that of GIDCSR.

**Figure 22:**
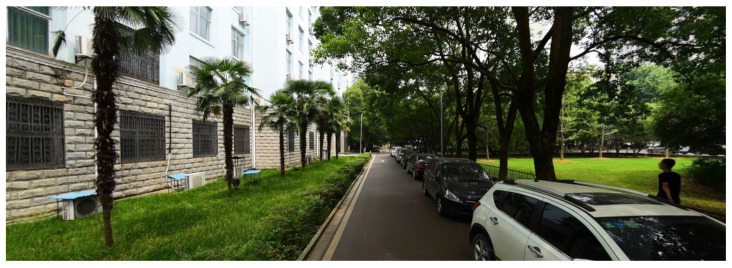
Scenario where vertical error of GIDCSR increases.

However, the vertical error shows the opposite comparison results. Affected by the geometric distribution of visible satellites, the error of GIDCSR increases quite much. In the corresponding time period, the vehicle went through a road beside a tall building (Figure 22). Almost all the satellites on the left were blocked. The poor performance of the GNSS only vertical positioning results also proved it (Figure 20a). Therefore, the degradation of the GIDCSR integration vertical positioning results is reasonable, as consequence. The vertical GNSS only positioning error is also large for the M8U observations post processing results (Figure 20a). However, the height errors of the integration results for M8U are small. Here it is worth noting that the horizontal positioning error of M8U integration results is dominated by the GNSS only results, but the height is basically unaffected by the GNSS. The reasonable explanation is that some vertical constraint, strong filtering, or dynamic prediction for height might be applied in M8U navigation engine, which improved the vertical positioning accuracy.

## 5. Conclusions and Future Work

A GNSS/INS deeply coupled software defined receiver (GIDCSR) with a low cost MEMS IMU is designed and implemented in this paper. Targeting the GNSS-challenged urban environments, two key technologies were proposed—adaptive open-close loop strategy and INS aided pseudo-range weight control algorithm. While the loop strategy is to improve the continuity and accuracy of the signal tracking, the weight algorithm can accurately estimate the pseudo-range gross error due to signal attenuation, blocking or reflection. To evaluate the performance of the proposed GIDCSR, a real-world field test in urban environment was carried out. The adaptive open-close tracking loops in the deep couple ensure carrier phase valid in the open sky, and improve pseudo-range accuracy before and after GNSS signal blocked. The INS aided pseudo-range weight control decreases the pseudo-range gross errors caused by multipath to improve the NCO control quality. Meanwhile, a typical GNSS/MEMS IMU couple product for vehicle navigation, u-blox M8U was compared. The pseudo-range error of GIDCSR is statistically 25.3% to 63.0% smaller than that of M8U, and GIDCSR outperforms M8U in terms of continuity. The GIDCSR’s horizontal positioning errors are 69% smaller than M8U’s. As a conclusion, with the advantages of the INS aiding for GNSS signal processing and pseudo-range weighting, the proposed GNSS/INS deep couple solution is capable of providing superior pseudo-range observations and more accurate horizontal positioning results in urban environments.

As a future work, the GNSS signal tracking, measurement generation and positioning based on carrier phase will be implemented in the GIDCSR for urban environments, which will significantly improve the performance of GIDCSR with precise positioning capability to meet the requirement of autonomous vehicles.

## Figures and Tables

**Table 1:** M8U IMU and ICM-20602 static AVAR. analysis parameters.

Parameter	M8U IMU	ICM-20602
Gyroscope angularrandom walk (deg/sqrt(hr))	0.48	0.23
Gyroscope bias instability (deg/hr)	10.8	10.7
Accelerometer velocityrandom walk (m/s/sqrt(hr))	0.06	0.042
Accelerometer bias instability (mGal)	50	16

**Table 2:** Statistical analysis before and after applying INS Aided Pseudo-range Weight Control Algorithm (IWA).

	SD(m)		Max(m)	
**Satellite PRN**	**IWA Enabled**	**IWA Disabled**	**IWA Enabled**	**IWA Disabled**
GPS 8	0.7793	1.3742	9.4010	15.3010
GPS 9	4.5917	7.4000	42.1690	65.4270
GPS 16	4.2423	10.1625	39.5030	84.2200
GPS 18	1.1373	1.7913	16.8610	23.8980
BD 10	0.7913	1.2251	22.7740	25.0350
BD 1	0.9583	2.5315	12.1420	16.3540
BD 29	0.8127	1.1714	19.4810	21.4100
BD 2	2.8286	3.1086	49.9910	51.5310

**Table 3:** GPS Satellites pseudo-range measurement performance (GIDCSR v.s u-blox M8U).

	SD(m)		Max(m)		Valid Epochs Proportion(%)	
GPS PRN	GID CSR	M8U	GID CSR	M8U	GID CSR	M8U
1	1.5972	2.355	10.176	13.634	98.93 %	93.11 %
8	0.7883	1.6264	9.527	15.792	100.00%	99.55%
9	4.3035	6.3829	42.269	64.313	86.31%	71.20%
11	0.8495	1.7414	8.892	14.208	100.00%	99.64%
16	3.9518	5.2928	40.428	35.55	85.15%	76.48%
18	1.1303	1.7902	16.94	21.105	100.00%	99.28%
27	1.8361	2.7757	23.151	27.899	99.11%	91.50%
30	1.5508	3.3141	22.371	60.433	97.32%	92.93%

**Table 4:** BD Satellites pseudo-range measurement performance (GIDCSR v.s u-blox M8U).

	SD(m)		Max(m)		Valid Epochs Proportion(%)	
BD PRN	GID CSR	M8U	GID CSR	M8U	GID CSR	M8U
1	0.9379	2.5351	12.149	23.299	99.11%	97.14%
2	2.6782	4.3748	49.553	62.227	78.80%	83.18%
3	1.098	1.5951	14.755	19.973	99.02%	98.03%
4	2.4376	4.1498	30.842	43.646	88.64%	81.04%
10	0.7915	1.592	22.765	27.779	99.46%	100.00%
29	0.804	1.586	19.457	26.105	99.73%	99.91%

**Table 5:** Statistics of GNSS positioning errors.

		GIDCSR	u-blox M8U
N	SD(m)	0.8878	1.1457
	Max(m)	4.1221	6.9427
E	SD(m)	1.3233	2.1456
	Max(m)	6.4749	12.5989
D	SD(m)	3.9818	6.4604
	Max(m)	24.9116	40.4575

**Table 6:** Statistics of GNSS/INS integration errors.

		GIDCSR	M8U
N	SD(m)	0.9686	2.8252
	Max(m)	3.4498	9.4681
E	SD(m)	1.2232	3.9018
	Max(m)	4.3111	11.5587
D	SD(m)	3.1172	1.8053
	Max(m)	11.2538	5.294
